# Non-accidental Trauma Masquerading As Feeding Intolerance in a Six-Month-Old Female

**DOI:** 10.7759/cureus.72788

**Published:** 2024-10-31

**Authors:** Rafael A Garcia, Orlando Torres

**Affiliations:** 1 Surgery, Ponce Health Sciences University, Ponce, PRI; 2 Pediatric Neurology, Ponce Health Sciences University, Ponce, PRI

**Keywords:** feeding intolerance, femur fractures, non-accidental trauma, pediatrics, spica cast

## Abstract

A six-month-old girl presented with feeding intolerance, fever, and left leg swelling. Imaging revealed a proximal left intertrochanteric fracture, along with rib and tibia fractures, raising concerns for non-accidental trauma. Blood cultures confirmed bacteremia caused by ceftriaxone-resistant *Serratia marcescens*, necessitating a 10-day course of antibiotics. The patient was immobilized with a hip spica cast, and metabolic and genetic evaluations were negative. She was discharged with instructions for follow-up care. This case highlights the importance of thorough evaluation for non-accidental trauma in infants presenting with unexplained fractures and systemic infections.

## Introduction

Neonates presenting with feeding intolerance to the emergency department present a complex diagnostic challenge due to the diverse array of potential underlying causes [1]. Feeding difficulties in this population can arise from gastrointestinal disorders, metabolic abnormalities, sepsis, and even non-accidental trauma (NAT). In this case, feeding intolerance refers to the infant's refusal to feed rather than gastrointestinal symptoms like vomiting. Recognizing the possibility of NAT is crucial, as its failure to be identified can have devastating consequences for the child [2].

Under The Child Abuse Prevention and Treatment Act (CAPTA), child abuse and neglect encompass acts or failures that result in serious physical or emotional harm or pose an imminent risk of harm. Musculoskeletal injuries, including fractures, are the second most common physical manifestations of NAT in children, following soft tissue injuries and bruising. Approximately 10% to 70% of children with NAT exhibit musculoskeletal injuries, with 30% to 50% of abused children seeking orthopedic care. Common etiologies behind NAT include physical abuse, neglect, and shaken baby syndrome. Prompt recognition of NAT is paramount, relying on obtaining a thorough history, including the child’s feeding patterns and caregiver behaviors. A comprehensive physical examination is also essential, with attention to signs of physical abuse, such as inconsistent bruising patterns and fractures in different stages of healing. Failure to report suspected abuse may result in recurrence in 30% to 50% of cases, with a 5% fatality rate [3]. Families will not often present twice to the same emergency room, so there may be only one chance to report the injury. Given the importance of recognizing potential indicators of child abuse, this case report aims to present a unique and previously unreported case of a six-month-old girl with NAT masquerading as feeding intolerance.

This article was previously presented as a meeting abstract at the 18th PRI/PHSU Annual Scientific Conference and Second RCMI Symposium in Health Disparities on May 6th, 2023.

## Case presentation

A six-month-old girl was brought to the emergency department due to progressive feeding intolerance, fever, and increasing irritability over several days. The feeding intolerance was characterized by refusal to feed rather than vomiting, which suggested the presence of discomfort likely related to fractures and systemic infection.

On physical examination, the left leg appeared swollen, particularly around the hip and thigh, with external rotation and restricted movement. Neurovascular examination was normal with intact pedal pulses bilaterally.

**Figure 1:**
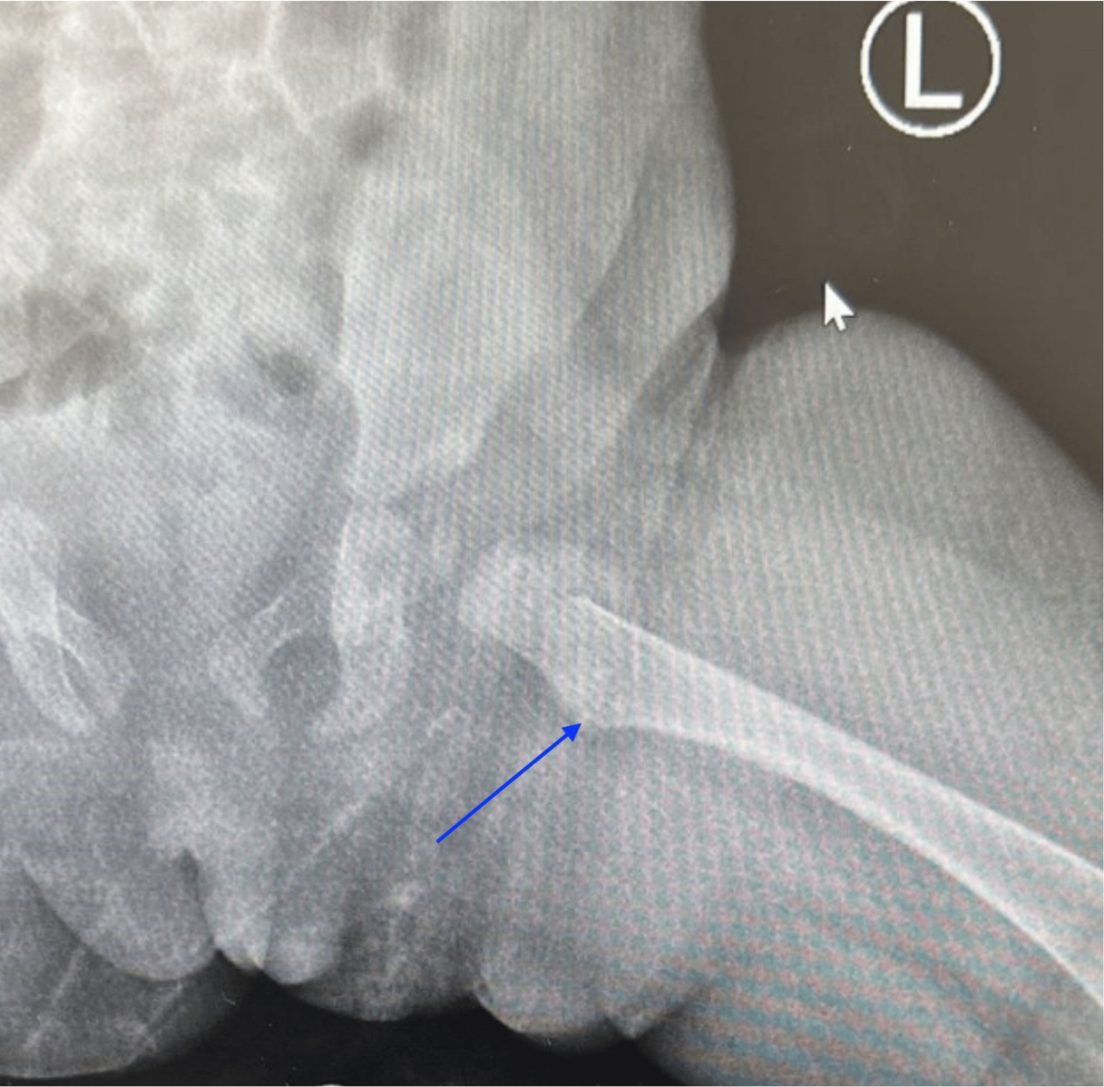
Proximal left femoral intertrochanteric fracture on radiograph of the patient.

**Figure 2:**
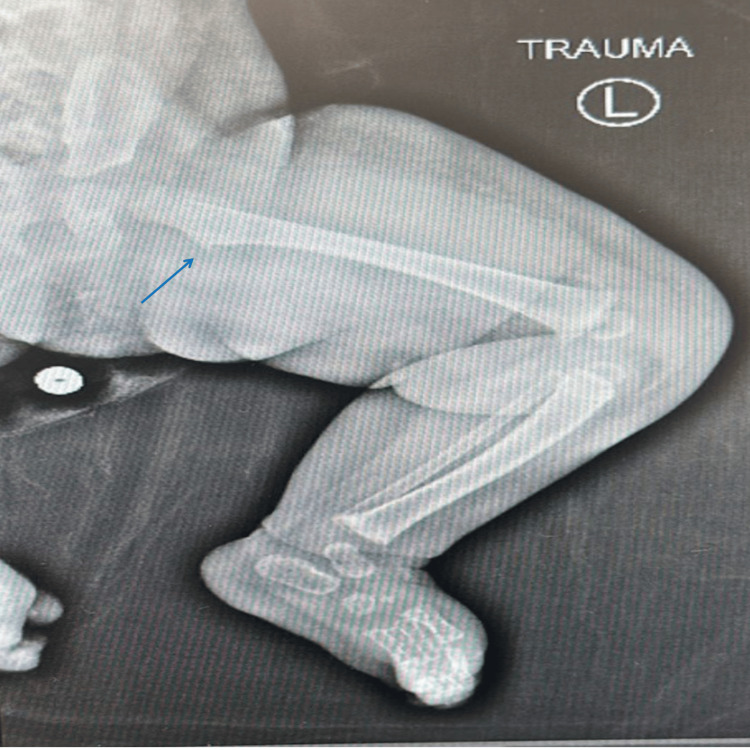
Proximal left femoral intertrochanteric fracture on radiograph of the patient.

An initial X-ray of the left lower extremity revealed a proximal left intertrochanteric fracture (Figures 1, 2). Given the patient’s age and the absence of a traumatic event, a full skeletal survey was conducted, revealing a left posterior rib fracture and a right tibia midshaft fracture, raising concern for NAT. Given the pattern of injuries, potential causes of NAT, such as physical abuse, were considered, particularly in the absence of an adequate explanation for the fractures.

Blood cultures were obtained due to the presence of fever, and results confirmed bacteremia caused by a ceftriaxone-resistant strain of *Serratia marcescens*. The patient was treated with intravenous ceftriaxone-resistant *Serratia marcescens* using a 10-day course of antibiotics. A metabolic and genetic workup was performed, yielding no abnormalities to suggest an underlying bone pathology.

The child was immobilized with a hip spica cast, and a multidisciplinary team was involved to evaluate potential child abuse further. After stabilization of the fractures and completion of the antibiotic course, the patient’s feeding difficulties resolved, and she was able to feed normally prior to discharge.

Following stabilization, the patient was transferred to the Family Department, and arrangements were made for child protective services to follow up. She was discharged with instructions to return for an orthopedic follow-up in two weeks to assess the spica cast and further fracture healing.

## Discussion

Child abuse, including NAT, presents significant challenges to healthcare providers due to its often subtle and varied presentations. While NAT is prevalent across socioeconomic backgrounds, the true incidence is likely underestimated due to challenges in data collection and underreporting [1,2]. This case highlights the importance of maintaining a high index of suspicion for NAT in pediatric patients presenting with unexplained fractures and systemic infections, such as bacteremia [3,4].

In this case, the patient presented with feeding intolerance, fever, and multiple fractures, all of which were initially unexplained. The differential diagnosis included multifocal osteomyelitis, which was ruled out based on the absence of radiographic evidence of osteomyelitis and the fact that the fractures were at different stages of healing. This pattern is more consistent with non-accidental trauma [5]. Additionally, the bacteremia caused by ceftriaxone-resistant *Serratia marcescens* added complexity to the case, but it was managed successfully with a 10-day course of antibiotics.

Prompt recognition and management of NAT are critical, as repeated abuse has a high risk of recurrence and severe long-term consequences [6]. A multidisciplinary approach involving orthopedic surgeons, pediatricians, and social services is essential for diagnosing NAT and ensuring the safety and well-being of the child. This case underscores the need for healthcare professionals to carefully evaluate the entire clinical picture, including behavioral signs like feeding intolerance, and to consider NAT when other explanations are lacking [3,7].

The clinical course of this patient’s recovery was favorable following fracture stabilization and antibiotic treatment. After applying a spica cast and the resolution of the bacteremia, the patient’s feeding difficulties improved, and she was discharged with a follow-up plan for fracture healing. Early recognition and intervention in cases of NAT can prevent further harm and promote better outcomes for vulnerable pediatric patients [2,7].

## Conclusions

This case underscores the critical importance of considering NAT in the differential diagnosis when evaluating an infant with progressive feeding intolerance, irritability, and unexplained fractures. Early identification and intervention are essential to protect vulnerable children and prevent further harm. A comprehensive clinical approach, obtaining a thorough history, performing a detailed physical examination, and considering a multidisciplinary evaluation differentiate NAT from other conditions, such as multifocal osteomyelitis. By maintaining a high index of suspicion and recognizing subtle signs of abuse, healthcare providers can ensure timely interventions that may be lifesaving. Raising awareness of these atypical presentations, such as feeding intolerance linked to physical trauma, will contribute to better outcomes and safeguarding the well-being of pediatric patients.
